# Comparison of surgical and obstetric outcomes in women with uterine leiomyomas after laparoscopic vs. abdominal myomectomy: A single-center cohort study

**DOI:** 10.3389/fsurg.2022.997078

**Published:** 2022-12-26

**Authors:** Polán Ordás, Emanuela Spagnolo, Lucía Gómez-Lavín Fernández, María Dolores Diestro Tejeda, Pilar Lafuente, Patricia Salas, Ana Lopez Carrasco, María Carbonell, Alicia Hernández

**Affiliations:** ^1^ Department of Obstetrics and Gynecology, La Paz University Hospital, Madrid, Spain; ^2^ Department of Obstetrics and Gynecology, Consorci Sanitari Parc Taulí, Hospital de Sabadell, Barcelona, Spain; ^3^ Department of Obstetrics and Gynecology, Faculty of Medicine, Universidad Autónoma de Madrid, Madrid, Spain

**Keywords:** uterine leiomyoma, laparoscopy, myomectomy, obstetric outcome, uterine rupture

## Abstract

Our aim was to study the advantages, complications and obstetrical outcomes of laparoscopic myomectomy (LM) compared with abdominal myomectomy (AM). We conducted a retrospective cohort study at La Paz University Hospital that included LMs and AMs performed between 2012 and 2018, analyzing 254 myomectomies (142 AMs [55.7%] and 112 LMs [43.9%]). The mean number of fibroids was 1.8 ± 1.5 and 3 ± 2.9 for the LM and AM groups, respectively (*p* < 0.006). The mean size of the largest myoma was 7.6 cm ± 2.7 cm and 10.2 cm ± 5.4 cm for the LM and AM groups, respectively (*p* < 0.001). LMs were associated with longer surgical times (*p* < 0.001) and shorter hospitalizations (*p* = 0.001). There were no significant differences in the intraoperative and postoperative complication rates (*p* = 0.075 and *p* = 0.285 for LM and AM, respectively). The subsequent pregnancy rate was higher for the LM group (30.8% vs. 16.8%, *p* = 0.009), with a vaginal delivery rate of 69% and no cases of uterine rupture.

## Introduction

Uterine fibroids, or leiomyomas, are benign uterine neoplasms that arise from smooth-muscle tissue and are present in 20%–40% of women of reproductive age (1–3) and 70%–80% of women older than 50 years (4, 5). In most cases, however, uterine fibroids go unnoticed, with only 40% of cases presenting symptoms. The most frequent symptoms are severe vaginal bleeding (and consequent anemia), pelvic pain, dysmenorrhea, worsening quality of life and reproductive dysfunction (6–9). The symptoms will depend basically on the composition, size, location and number of myomas (4–6, 9).

The classical treatment for uterine fibroids has been open abdominal hysterectomy. Cultural and social developments, as well as delayed conception age, have led to the development and improvement of myomectomy, which was first described in the 1970s. For those patients who wish to retain their reproductive ability, myomectomy is an alternative to hysterectomy (3).

The advent of minimally invasive techniques has significantly improved the short-term outcomes of major gynecologic surgery, including myomectomy, by enhancing recovery and decreasing pain and postoperative complications (10, 11). However, the current criteria for selecting patients and deciding on the surgical approach are still a matter of debate (10, 12).

The presence of leiomyomas can affect obstetrical outcomes, causing decreased fertility, increased pregnancy loss and complications during pregnancy (13–15). The delivery mode for these patients is controversial, given that many obstetricians recommend elective caesarean delivery for most patients with a previous history of myomectomy (mainly if the uterine cavity was entered at the time of surgery), despite a lack of strong supporting evidence (15).

Therefore, this study's main objective was to compare the surgical and obstetrical outcomes of laparoscopic myomectomy (LM) vs. open abdominal myomectomy (AM), thereby establishing selection criteria for the most appropriate surgical approach.

## Materials and methods

After institutional review board approval (PI-3661), this retrospective cohort study included all patients who underwent LM or open AM at the Department of Gynecology of La Paz University Hospital between May 2012 and December 2018. The study included patients aged 18–50 years, with an ultrasound diagnosis of at least 1 myoma with a mean diameter ≥3 cm and presenting heavy menstrual bleeding or infertility, pelvic pain or suspicious ultrasound findings (16) as the main indications for myomectomy. The exclusion criteria were a postmenopausal state, history of primary ovarian insufficiency or tubal factor infertility and the presence of uterine mass suspicious for malignancy. Patients were grouped according to the surgical approach: LM group and AM group.

We collected preoperative data that included age, body mass index, indication for surgery, preoperative hemoglobin levels, previous myomectomy, previous use of ulipristal acetate (5 mg daily for 3–6 months), total number of myomas and the diameter and location of the largest myoma as determined by ultrasound. A preoperative systematic ultrasound examination was conducted in all cases, classifying the fibroids according to the International Federation of Gynecology and Obstetrics classification (17). We analyzed the details of the surgery, hospital stay and histology and described and compared the intraoperative and postoperative complications according to the Clavien-Dindo classification (18), as well as the recurrence rates. Lastly, we investigated the pregnancy rates, conception method, delivery type and delivery outcomes.

### Surgical technique

Myomectomy was performed in both groups using the intracapsular technique with pseudocapsule preservation, as previously described (19). The surgeries were performed by an expert (more than 10 years of experience) minimally invasive gynecologic surgeon (Table 1) or training surgeon. To reduce intraoperative bleeding during the laparoscopic myomectomy, the patients were administered an intramyometrial injection of dilute vasopressin (20 IU/100 ml normal saline), or a temporary uterine artery occlusion was performed using vascular clips (10-mm Hem-o-lok clip or medium titanium clip). LM was performed using a 10-mm trocar for the camera (placed at or above the umbilicus, depending on the size of the uterus). Two ancillary 5-mm trocars were inserted in each iliac fossa, and one ancillary 10–12 mm trocar was inserted in the suprapubic area. For the LM group, a RUMI® II System uterine manipulator (Cooper Surgical, Inc, Trumbull, United States) was employed. The surgeon therefore performed the following steps: transversal or oblique hysterotomy using a crochet needle electrode, enucleation of the myoma (by traction and counter-traction movements using a strong grasper and an irrigator cannula inserted in the space under the myoma pseudocapsule and fibroid) and suture of the uterine defect. A bipolar clamp was used for selected hemostasis. The myoma was finally extracted from the abdominal cavity using manual morcellation through the umbilical incision in endobag. The uterine walls were sutured in 1 or 2 layers, according to the depth of the hysterotomy, with a continuous suture. For the uterine suture, the surgeon employed a size 0 barbed suture (VLock, Covidien) or an absorbable monofilament thread 1/0 (Biosyn, Covidien).

**Table 1:** Patient characteristics of the two groups (laparoscopic myomectomy versus abdominal myomectomy).

	LM (*n* = 112)	AM (*n* = 142)	Total (*n* = 254)	*p*
**Age (years)**	35.91 (±5.517)	37.17 (±4.902)	36.60 (±5.244)	0.057
**BMI (kg/m^2^)**	23.37 (±4.631)	24.30 (±4.800)	23.82 (±4.749)	0.129
**Preoperative Hb (g/dl)**	13.15 (±1.279)	13.15 (±1.358)	13.15 (±1.328)	0.991
**Barriers myomectomy**	6 (5.4%)	12 (8.4%)	18 (7.2%)	0.310
**Use of ulipristal acetate**	15 (13.4%)	18 (12.7%)	33 (13%)	0.935
**Indication**	* *	* *	* *	0.886
Several vaginal bleeding	35 (31.3%)	50 (35.2%)	85 (33.5%)	
Abnormal growing	31 (27.7%)	34 (23.9%)	65 (25.6%)	
Pelvic pain	25 (22.3%)	31 (21.8%)	56 (22%)	
Infertility	21 (18.8%)	27 (19%)	48 (18.9%)	
**Ultrasound description**				
** Number of myomas**	1.54 (±1.472)	1.99 (±2.076)		**0.022**
** Largest size (cm)**	6.79 (±2.175	8.67 (±2.631)		**0.000**
** Type of the largest**				0.567
Pedunculated	15 (13.4%)	10 (7.1%)		
Subserous	28 (25%)	30 (21.1%)		
Subserous-intramural	24 (21.4%)	29 (20.4%)		
Intramural	44 (39.3%)	69 (48.6%)		
Intramural-submucous	1 (0.9%)	4 (2.8%)		
**Location of the largest**				0.791
Anterior	26 (23.2%)	34 (23.9%)		
Posterior	49(43.7%)	52 (36.6%)		
Fundus	16 (14.3%)	23 (16.2%)		
Right	8 (7.1%)	16 (11.3%)		
Left	9 (8%)	12 (8.4%)		
Other	4 (3.6%)	5 (3.5%)		
**FIGO type of the largest**				0.113
2	0	3 (2.1%)	3 (1.2%)	
3	3 (2.7%)	5 (3.5%)	8 (3.1%)	
4	27 (24.1%)	20 (14.1%)	47 (18.5%)	
5	32 (28.6%)	53 (37.3%)	85 (33.5%)	
6	37 (33%)	52 (36.6%)	89 (35%)	
7	13 (11.6%)	9 (6.3%)	21 (8.3%)	
**Surgical data**				
**Number of myomas removed**	1.79 (±1.51)	3.07 (±2.86)		**0.006**
**Size of the largest myoma (cm)**	7.55 (±2.73)	10.24 (±5.42)		**<0.001**
** Procedures to reduce intraoperative bleeding**				
None	65 (58%)	–	–	
Uterine artery occlusion (clips)	26 (23.2%)	–	–	
Vasopressin	21 (18.8%)	–	–	
**Uterine cavity disruption**	20 (17.9%)	30 (19.7%)		0.559
**Operating time (min)**	147.38 (±63.79)	95 (±37.47)		0.001
**Additional procedure****^†^**	19 (17%)	13 (9.1%)		0.450
**Expert surgeon****^‡^**	103 (92%)	48 (33.8%)		0.001
**Use of anti-adherents**	42 (37.5%)	50 (35.2%)		0.885
**Hospital stay (days)**	4.57 (±2.1)	5.49 (±1.13)		0.001
**Histology**				0.278
Typical leiomyoma	106 (94.6%)	138 (97.1%)	244 (96.1%)	
Leiomyoma with bizarre nuclei	3 (2.7%)	4 (2.8%)	7 (2.7%)	
Adenomyoma	2 (1.8%)	–	2 (0.8%)	
STUMP	1 (0.9%)	–	1 (0.4%)	

Values are *n* (%) or mean ± standard deviation. LM, laparoscopic myomectomy; AM, abdominal myomectomy; BMI, body max index; Hb, hemoglobin; STUMP, smooth muscle tumor of uncertain malignant potential.

^†^ Including: ovarian cystectomy, salpingectomy, appendectomy, bowel resection for deep endometriosis and resection endometriosis lesions.

^‡^ >10 years of experience.

The AM procedure was performed by a mini-Pfannenstiel incision. The surgeon attempted to remove all visible myomas by using a minimum number of incisions. The uterine defect was repaired using an absorbable multifilament thread 1/0 (Vicryl, Ethicon; Johnson & Johnson) or an absorbable monofilament thread 1/0 (Biosyn, Covidien). As previously described for the LM group, the uterus was sutured in 1 or 2 layers, according to the depth of the hysterotomy, with a continuous suture.

In both surgical techniques, the pelvis was irrigated with saline solution at the end of the procedure. For both groups, the use of absorbable adhesion barrier (oxidized regenerated cellulose; Surgicel, Ethicon) and drain was not standardized, and they were placed only if the surgeon considered them necessary.

### Statistical analysis

The statistical analysis was performed using SPSS version 24.0 for Windows (IBM Corp., Armonk, NY, United States). The quantitative variables are expressed as mean and standard deviation, and the qualitative variables are expressed as absolute numbers and percentages. The quantitative variables were analyzed between the two groups using Student's *t*-test, whereas the qualitative variables were analyzed using the chi-squared test and Fisher's exact test. To assess the significance of the individual parameters, we performed a bivariate logistic regression analysis. To investigate the combination of predictors for the myomectomy route, we conducted a multiple regression analysis. To assess the degree of agreement between the ultrasound examination and the surgery evaluation, we calculated the intraclass correlation coefficient (ICC). A *p*-value <0.05 was considered statistically significant for all variables.

## Results

### Surgical outcomes

From May 2012 to December 2018, a total of 254 myomectomies were performed (112 [43.9%] LMs and 142 [55.7%] AMs). In 4 (3.6%) cases, the surgery started by laparoscopy and then converted to open surgery; these cases were therefore assigned to the AM group. In 2 of these cases, the conversion was due to the enormous size of the myoma (>15 cm), and in the other 2 cases, the conversion was due to massive blood loss.

Table 1 summarizes the patients' preoperative characteristics and surgical data, while Table 2 summarizes the intraoperative and postoperative complications, the latter of which were mostly minor and controllable with no invasive procedures. In contrast, 1 patient of the AM group (Table 2) underwent emergency abdominal hysterectomy due to uncontrolled intraoperative blood loss (>1000 ml). One (0.9%) case in the LM group and 5 (3.6%) cases in the AM group experienced recurrence and required reintervention, a difference that was not statistically significant (*p* = 0.285).

**Table 2:** Comparison of the intraoperative and postoperative complications between laparoscopic myomectomy and abdominal myomectomy.

	LM (*n* = 112)	AM (*n* = 142)	*p*
**Intraoperative complications**	6 (5.3%)	9 (6.3%)	0.075
Organ injury^†^	2 (1.8%)	5 (3.5%)	* *
Estimated blood loss >1,000 ml	1 (0.9%)	4 (2.8%)	0.559
**Postoperative complications** **^‡^**	17 (15.2%)	29 (20.4%)	0.336
Grade 1	3 (2.7%)	5 (3.5%)	* *
Grade 2	11 (9.8%)	20 (14.1%)	* *
Grade 3	2 (1.8%)	4 (2.8%)	* *
Grade 3a	0	0	* *
Grade 3b	2 (1.8%)	4 (2.8%)	* *
Grade 4	0	0	* *

Values are *n* (%). LM, laparoscopic myomectomy; AM, abdominal myomectomy.

**^†^**Including: bowel, uterus, bladder, ovaries, small intestine and ureter.

^‡^ Clavien-Dindo Classification system.

The logistic regression analysis demonstrated that the number of enucleated myomas, the size of the largest myoma and the surgeon's experience were related to the outcomes and could predict the success of the surgical approach. Our results showed that having more than 3 myomas reduced the probability of a successful LM by 69.3% (*p* = 0.001), which also applied to the size of the largest enucleated myoma. The probability of successful enucleation was 3-fold higher with laparotomic access than with laparoscopic access for myomas measuring >9 cm [odds ratio 3.24, 95% confidence interval (CI) 1.865–5.656] (*p* < 0.001). Moreover, the surgeon with more than 10 years of experience in laparoscopic surgery increased the chances of a successful minimally invasive surgery of uterine fibroids by 20,431 fold (95% CI 8704–47,956; *p* < 0.001).

Lastly, we calculated the ICC to determine whether there was a correlation between the number of myomas observed by ultrasound and the number of myomas observed during the surgical procedure. We obtained an ICC of 0.729 (95% CI: 0.591–0.812; *p* < 0.001), which indicated “high similarity”. The dimensions of the major myoma observed in the preoperative ultrasound showed moderate agreement with those ultimately observed during surgery (ICC: 0.686; 95% CI: 0.597–0.756; *p* < 0.001).

### Obstetric outcomes

**Figure 1:**
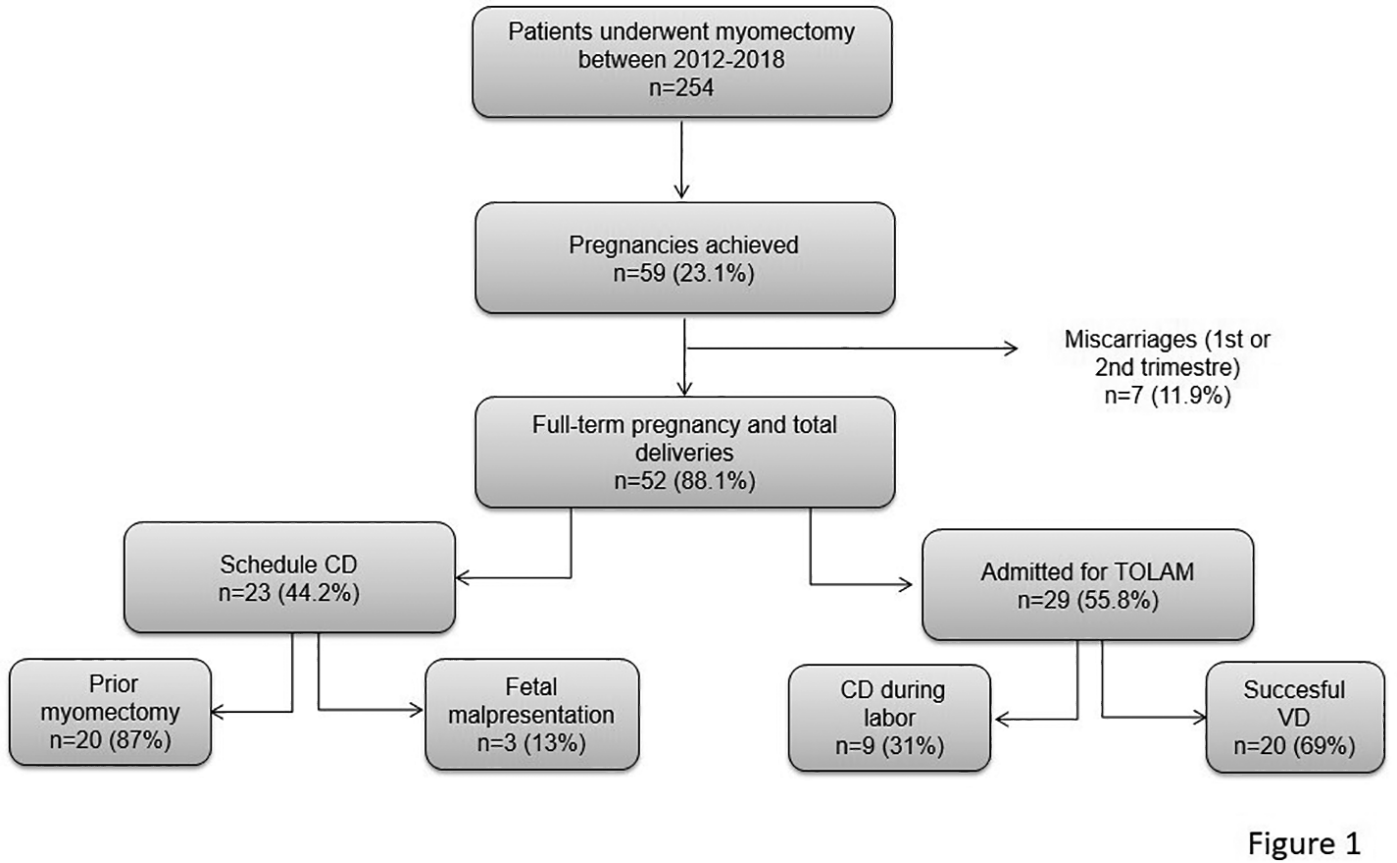
Flow diagram of study population: pregnancy and delivery outcomes in patients who underwent laparoscopic and abdominal myomectomy. CD, cesarean delivery; TOLAM, trial of labor after myomectomy; VD, vaginal delivery.

Table 3 shows the obstetric outcomes of the 2 groups, and Figure 1 summarizes the pregnancy and delivery outcomes. The overall pregnancy rate (pregnancies with evidence of a viable fetus) for the patients whose indication for surgery was infertility was 40% (23 patients), compared with the other indications: 16.9% (10 patients) for vaginal bleeding, 25.4% (15 patients) for suspicious ultrasound findings and 18.6% (11 patients) for pelvic pain (*p* < 0.001). Moreover, in the patients whose indication for surgery was infertility, a higher pregnancy rate was maintained in the LM group than in the AM group (61.9% [13/21 patients] vs. 37% [10/27 patients]); however, the difference was not statistically significant (*p* = 0.087). There was an increase in the overall pregnancy rate (33%) for the patients whose largest enucleated myoma was <8 cm compared with those whose largest myoma was ≥8 cm (20%) (*p* = 0.022), regardless of surgical approach. There were no cases of major maternal or obstetric complications such as preterm delivery, placental abnormalities or placental abruption in either group.

**Table 3:** Obstetrics and delivery outcomes according surgical approach.

	LM (*n* = 36/112)	AM (*n* = 23/142)	TOTAL (*n* = 59/254)	*p*
**Pregnancy rate**	36 (30.8%)	23 (16.2%)	59 (23.2%)	**0** **.** **009**
**Conception method**				0.159
Spontaneous pregnancy	26 (72.3%)	13 (56.5%)	39 (66.1%)	
ART (including IVF or AI)	10 (27.7%)	10 (43.5%)	20 (33.9%)	
**Pregnancy outcome**				0.680
Miscarriage	4 (11.1%)	2 (8.7%)	6 (10.2%)	
Intrauterine fetal death	1 (2.8%)	–	1 (1.7%)	
Full-term delivery	31 (86.1%)	21 (91.3%)	52 (88.1%)	
**Type of delivery**				0.074
Vaginal	15 (48.4%)	5 (23.8%)	20 (38.5%)	
Cesarean	16 (51.6%)	16 (76.2%)	32 (61.5%)	
	GROUP I:LM (*n* = 31)	GROUP II: AM (*n* = 21)	TOTAL (*n* = 52)	*p*-value
**Mode of delivery**				**0** **.** **035**
** Elected cesarean delivery**	10 (32.3%)	13 (61.9%)	23 (44.2%)	0.035
Prior myomectomy	8 (80%)	12 (92.3%)	20 (87%)	0.385
Fetal malpresentation	2 (20%)	1 (7.7%)	3 (13%)	0.385
** Admitted for TOLAM**	21 (67.7%)	8 (38.1%)	29 (55.8%)	0.035
Cesarean delivery during labor	6 (28.6%)	3 (37.5%)	9 (31%)	0.642
Failed induction of labor	1 (16.6%)	1 (33.3%)	2 (22.2%)	
Failure to progress	1 (16.6%)	1(33.3%)	2 (22.2%)	
Fetal distress	2 (33.3%)	1(33.3%)	3 (33.3%)	
Cephalopelvic disproportion	2 (33.3%)	–	2 (22.2%)	
Accomplished vaginal delivery in patients admitted for TOLAM	15 (71.4%)	5 (62.5%)	20 (69%)	0.074

Values are *n* (%). LM, laparoscopic myomectomy; AM, abdominal myomectomy; ART, assisted reproductive techniques; IVF, *in vitro* fertilization; AI, artificial insemination; TOLAM, trial of labor after myomectomy.

Our series presented a miscarriage rate of 10.2% and an overall full-term delivery rate of 88.1%. All deliveries resulted in live births, and there were no cases of uterine rupture or postpartum hemorrhaging in our series. The pregnancy outcomes of the 2 groups are summarized in Table 3.

## Discussion

Myomas are the most common female benign tumors and frequently require surgery. Although there are no universally accepted selection criteria for the surgical approach, based on our results, women with 3 or more fibroids and whose largest myoma is ≤9 cm can be successfully treated through laparoscopy. Our results agree with those of previous studies that showed that the number of complications, surgical difficulties and risk of laparoconversion increase if LM is performed in patients with these characteristics (11, 20–22).

A preoperative ultrasound evaluation should be systematically performed to select the most appropriate surgical approach because ultrasound provides information on the number, size, type and location of the fibroids (14, 21). Frascà et al. (2018) (23) concluded that preoperative ultrasound can correctly identify the number, type, size and locations of myomas with an accuracy >72%. Our results showed that there was a high correlation between the number and size of the largest myoma observed by ultrasound and those identified during surgery.

### Does LM improve surgical outcomes compared with AM?

In our series, the women who underwent LM and open AM were comparable in their preoperative characteristics. The association between the surgeon's experience and the laparoscopic approach was statistically significant (*p* < 0.001), with 92% of the laparoscopies performed by expert surgeons and 66% performed by training surgeons. The surgeon's experience continues to be decisive for successful LM (20, 21). We therefore found no association between the type of myoma and the surgical approach (*p* = 0.767), given the expert surgeon's skill in managing different types of myomas. For the same reason, there was no significant difference in the estimated blood loss between the groups (*p* = 0.559). Despite the surgeon's experience, LM required longer surgical times (*p* < 0.001), as previously reported (13, 22).

With regard to intraoperative and postoperative complications, we found no statistical differences between the 2 groups, as reported in the literature (13). In our study, the mean hospital stay was shorter for the LM group than for the AM group, corroborating the reports of previous studies (13, 24, 25), thereby confirming the major advantage of the laparoscopic approach. Furthermore, a recent meta-analysis (26) which compared transvaginal retrieval and port-site specimen retrieval after LM showed comparable results in terms of intraoperative complications, hospital stay and operative time. Concerning transvaginal extraction of the surgical specimen after LM, a large case series by Laganá et al. (27) demonstrated a significant increase in operative time, intraoperative blood loss and hospital stay with increasing weight of removed fibroid.

One of the limitations of the laparoscopic approach is the inability to palpate the uterus during the intervention, which can result in persistence of intramural fibroids (22). However, this limitation could be overcome by performing an intra-operative vaginal ultrasound to confirm the presence of any intramural fibroids prior to completing the surgery (23). Studies have established that the recurrence rates for patients who undergo LM are 167% higher than for those who undergo AM, with a cumulative 5-year recurrence rate of 57.3%–62.1% for the LM group and 15.4%–62% for the AM group (13, 28). Recent studies have reported that although recurrence rates after LM might be high, the number of patients who require re-operation is low (2.4%) (29, 30). Our study showed different results, given that 5 (3.62%) patients underwent re-operation after AM vs. only 1 after LM (0.9%), although there were no significant differences, and the reintervention rates were low in both cases. Lastly, it is important to highlight the effect of additional procedures such as endometriosis on the operative time. Although there were no statistical differences in operative times between the 2 groups, the association between endometriosis and myomas is very common. In particular, eradication of deep endometriosis could affect surgical times and could represent a selection bias in our study.

### Do obstetric outcomes improve after LM compared with AM?

Numerous studies have indicated that the cumulative pregnancy rate and cumulative live birth rate are similar between women treated by LM and those treated with AM (30, 31). Our study showed a higher pregnancy rate after LM (30.8% vs. 16.8%), which was statistically significant (*p* = 0.009). Our results agree with those of other studies that found that patients who underwent LM had a higher pregnancy rate than those who underwent AM, possibly due to a reduced occurrence of postoperative adhesions (14, 21). In our study, anti-adherent barriers were employed in 37.5% of the LM procedures and in 36.2% of the AM procedures. Although these differences are not statistically significant, we can speculate that their use in LM might prevent adhesions. In a recent review (32), oxidized regenerated cellulose was found to be effective in reducing the total adhesion score during second-look surgery. Laganá et al. (33) showed that the opening of uterine cavity and the laparotomic approach represented independent risk factors for developing intrauterine adhesions, after 3 months from surgery.

Unfortunately, it is difficult to speculate on the relationship between increased pregnancy rates among patients who underwent LM and those who underwent AM.

Numerous studies have shown that pregnancy rates increased up to 70% after myomectomy (14) due to various mechanisms, such as distortion of the uterine cavity, alteration of myometrial contractility and alteration of the tube-ovary anatomic relationship (34). Our data showed a higher pregnancy rate for the patients whose indication for surgery was sterility compared with the other indications (40% vs. <25.4%, respectively; *p* < 0.001), Furthermore, the gestational rate was higher for the LM group than for the AM group (61.9% vs. 37%), although these clinical differences were not statistically significant (*p* = 0.087). Our results showed a higher pregnancy rate (33% vs. 20%) among the patients whose main excised myoma measured <8 cm (*p* = 0.020), regardless of the surgical approach. Similar results were reported by Kundu et al. (2018) (14), who indicated a pregnancy rate of nearly 50% in the group with removed myomas <8 cm in diameter, dropping to ≤30% when the myomas measured ≥8 cm in diameter.

With regard to delivery outcomes, the elective caesarean rate was 44.2% in our series, with a higher rate of scheduled caesareans in the AM group than the LM group (69.1% vs. 32.3%; *p* = 0.035). The main indication for scheduling a caesarean in our study was opening the uterine cavity and performing multiple myomectomy (85% of cases) to prevent uterine rupture during labor. Our results agree with those of Gambacorti-Passerini et al. (2018) (15), who also reported a higher rate of scheduled caesareans in their AM group (75% vs. 46.7% in the LM group), with the main indication for caesarean being a previous myomectomy. The authors compared the operative findings of the women admitted for trial of labor after myomectomy (TOLAM) with those who were scheduled for caesareans and found no significant differences apart from the incidence of entering the uterine cavity (33.3%) in the caesarean group compared with the patients admitted for TOLAM (1.4%; *p* < 0.01).

Surprisingly, a recent meta-analysis by Claeys et al. (2014) (35) demonstrated that LM is associated with a higher rate of elective caesareans (*p* = 0.001), which contrasts with our results and those of previous studies. The authors attributed the higher rate in the LM group to the surgeon's concerns that this type of myomectomy has a higher risk of rupture during subsequent pregnancies and labor compared with the open technique. Interestingly, the URIDA (uterine rupture international data acquisition) study (19), did not showed any association between the incidence of uterine rupture and the number and type of previous uterine surgeries. This study evaluated 224 patients with previous uterine operations (cesarean section, LM, AM, hysteroscopic myomectomy). In these patients, the mean gestational age at which the uterine rupture was 37.32 ± 5.09 weeks.

In our study, 21 (67.7%) patients in the LM group and 8 (38.1%) patients in the AM group (*p* = 0.035) were admitted for TOLAM. Fifteen (71.4%) patients achieved a successful vaginal delivery after LM, and 5 (62.5%) patients achieved it after AM (*p* = 0.074). Gambacorti-Passerini et al. (2018) (15) and Claeys et al. (2014) (35) showed similar results, with a vaginal delivery rate of 91.5% for the patients admitted for TOLAM, 93% after LM and 88% after AM, with no statistically significant difference between the two surgical approaches. Although our findings showed a lower percentage of vaginal delivery by the patients admitted for TOLAM in both types of myomectomy than in prior studies, we had no cases of intrapartum complications and a lower rate of caesarean deliveries. Our results are therefore encouraging. Considering the aforementioned data, we believe TOLAM should be considered a feasible and safe option in tertiary level hospitals for patients with a history of myomectomy.

We had no cases of uterine rupture in our series, a complication that represents the main obstetrical risk for women with a previous myomectomy. Uterine rupture is rare (0.47%–1%) and difficult to predict (15, 20, 36).

The strength of our study was its large patient cohort and the analysis of numerous factors in addition to surgical variables. Our study is limited by the retrospective data collection, which might have prevented us from collecting more exact data on cofactors that might have influenced the attitude of obstetricians in determining the delivery mode.

## Conclusion

As an alternative to AM, LM can be considered a safe and suitable surgical technique for women of childbearing age, although LM requires longer surgical times and should be performed by surgical teams with a high degree of expertise and experience. A preoperative ultrasound evaluation of the size and number of myomas should be performed for careful patient selection. In our practice, LM was successfully performed in cases with ≤3 fibroids in which the largest measured ≤9 cm. The delivery mode for patients with a prior myomectomy should be individually established; however, vaginal delivery after LM should be considered a safe option for these patients, with uterine rupture an extremely rare complication.

## Data Availability

The original contributions presented in the study are included in the article/Supplementary Material, further inquiries can be directed to the corresponding author.
